# Assessment of the Efficacy and Pulmonary Toxicity of Trastuzumab Deruxtecan in HER2-Positive and HER2-Low Metastatic Breast Cancer in a Tertiary Center in the United Arab Emirates

**DOI:** 10.7759/cureus.78471

**Published:** 2025-02-04

**Authors:** Mohammad Hourani, Rawan Bdair, Emad Dawoud, Diaeddine A Trad, Selvaraj Giridharan, Khaled Al Qawasmeh, Husam Marashi, Jawaher Ansari

**Affiliations:** 1 Department of Oncology, Tawam Hospital, Al Ain, ARE; 2 Department of Genetics and Genomics, United Arab Emirates University, Al Ain, ARE

**Keywords:** her2-low expression, her2-positive, interstitial lung disease, metastatic breast cancer, trastuzumab deruxtecan

## Abstract

Introduction: Trastuzumab deruxtecan (T-DXd) is a HER2-directed antibody-drug conjugate indicated for the treatment of unresectable or metastatic HER2-positive breast cancer in patients who have received a prior anti-HER2-based regimen. T-DXd is also indicated for unresectable or metastatic HER2-low breast cancer, following prior chemotherapy in the metastatic setting or recurrent disease within six months of adjuvant chemotherapy. This study aims to evaluate the efficacy and safety of T-DXd in treating HER2-positive and HER2-low metastatic breast cancer (MBC) patients in a real-world clinical setting. In the T-DXd pivotal research, imaging assessments were conducted every six weeks with CT or MRI, but outside of a clinical trial setting, this frequent imaging is practically challenging due to resources and difficulty in reimbursing. In addition to clinical outcome assessments, we sought to review the incidence of pneumonitis in a real-world setting and to assess if the three-monthly response assessment scans would be sufficient to rule out asymptomatic pneumonitis.

Methods: A retrospective analysis was conducted on 100 patients diagnosed with HER2-positive (immunohistochemistry (IHC) 3+ or in situ hybridization (ISH) positive) or HER2-low (IHC 1+ or IHC 2+ and ISH negative) MBC treated with T-DXd at 5.4 mg/kg every 21 days, with standardized dose adjustments as required. Treatment was continued until disease progression or unacceptable toxicity. The median follow-up duration was 15 months. Responses were assessed using the Response Evaluation Criteria in Solid Tumors (RECIST) v1.1 criteria, and toxicity was determined using the Common Terminology Criteria for Adverse Events (CTCAE) version 5.0. Data analysis was performed using SPSS IBM software version 26 (IBM Corp., Armonk, New York, NY, US).

Results: The median age of the patients was 47 years, ranging from 29 to 86 years, with the majority being younger than 65 and predominantly women. All patients had an Eastern Cooperative Oncology Group (ECOG) performance status of 0-1 at baseline. Most patients were HER2-positive, while a smaller proportion were classified as HER2-low. A significant number of patients presented with visceral disease, while a smaller subset had brain metastases at the time of baseline evaluation. Nearly half of the patients were hormone receptor-positive (HR+), and more than half had a high Ki-67 index of over 20%. The majority of patients received T-DXd as a second- or third-line treatment. Clinical responses included partial response, complete response, and stable disease. Survival outcomes showed high overall survival rates at 12 and 24 months, with a median progression-free survival (PFS) of 24 months. Only one patient experienced grade 3 pneumonitis, suggesting that 12-weekly imaging assessments may be adequate for monitoring most patients.

Conclusions: Our real-world experience confirms that the efficacy of T-DXd in UAE is consistent with published data from published phase 3 clinical trials. The incidence of clinical pulmonary toxicity is much lower than anticipated, and until further data is available, it may be reasonable to continue using the 12-weekly assessment scans to monitor patients for interstitial lung disease (ILD). Further studies are needed to determine the optimal imaging frequency for monitoring ILD.

## Introduction

Breast cancer, the most common cancer globally, represents 12.5% of all new cancer cases each year and significantly impacts millions of lives [1]. Its incidence is projected to rise substantially, with estimates suggesting 3.2 million by 2050 [2]. In the UAE, breast cancer constituted 21.4% of all cancer cases in 2020, highlighting its regional impact [3]. Recent advances in molecular profiling using DNA microarrays have identified distinct subtypes of breast cancer, each with unique biological and clinical characteristics [4]. These subtypes have revolutionized breast cancer treatment and management [5,6]. Despite these advancements, metastatic breast cancer (MBC) remains incurable, though targeted therapies have improved survival rates [7].

Human epidermal growth factor receptor 2 (HER2), a member of the epidermal growth factor receptor (EGFR) family, contains 1,255 amino acids and is located on chromosome 17q21.1. While HER2 is expressed at low levels in normal tissues, its overexpression is associated with uncontrolled cell proliferation, leading to cancer development [8]. HER2-positive is defined by either a score of 3+ via immunohistochemical (IHC) analysis or through positive results in in situ hybridization (ISH). HER2-low status is defined through a score of +1 or +2 on IHC analysis and negative results in ISH [9]. HER2 receptor overexpression is observed in approximately 20%-30% of invasive breast carcinomas, driving downstream signaling pathways that promote tumor cell proliferation and survival [10,11]. As a result, HER2 is associated with more aggressive disease and a poorer prognosis, making it a key factor in clinical decision-making and patient management [12].

The development of targeted therapies such as trastuzumab, pertuzumab, and lapatinib has significantly improved the outcomes of HER2-positive breast cancer patients [13,14]. However, drug resistance to HER2 inhibitors remains challenging, necessitating the identification of new novel therapeutic targets [15]. Antibody-drug conjugates (ADCs) are biopharmaceutical drugs that have revolutionized cancer treatment and other diseases by enabling the direct delivery of potent cytotoxic drugs to cancer cells while minimizing exposure to healthy tissues [16,17]. The mechanism of action of ADCs involves binding to specific antigens expressed on cancer cells' surface, releasing a cytotoxic drug, and ultimately causing cell death. These ADCs are particularly useful for cancers with identifiable biomarkers, with each ADC designed to target a specific antigen overexpressed in particular types of cancer [18,19]. An example of this approach is trastuzumab deruxtecan (T-DXd): an anti-HER2 human monoclonal antibody, trastuzumab, linked to a topoisomerase I inhibitor, deruxtecan DXd, as its payload [19]. T-DXd has a higher drug-to-antibody ratio than previously approved TDM-1, possibly contributing to its enhanced efficacy [20]. The conjugation of DXd to trastuzumab via a maleimide linker sensitive to proteolysis has resulted in the formation of T-DXd with a homogeneous drug-to-antibody ratio [21]. The success of ADCs has transformed the treatment of HER2-positive breast cancer, with two FDA-approved agents, T-DXd and sacituzumab govitecan, showing efficacy. These agents have reenergized the field of ADC development and provide hope for patients with HER2-positive breast cancer [22,23].

## Materials and methods

This retrospective study was approved by the Tawam Human Research Ethics Committee (approval number KD/AJ/705). This study aims to evaluate the efficacy and safety of T-DXd in treating HER2-positive and HER2-low MBC patients in a real-world clinical setting, focusing on pulmonary toxicity after exposure to T-DXd. It included patients with MBC of either gender, aged 18 years or older, who were diagnosed between December 2021 and June 2024 at Tawam Hospital, UAE. Eligible patients had either HER2-positive or HER2-low MBC and had received one or more lines of systemic treatment prior to commencing T-DXd therapy. HER2-positive status was determined based on either a 3+ score from IHC analysis or positive ISH results, which were centrally verified against archival tissue samples. Patients classified as HER2-low were those with IHC scores of +1 or +2 and negative ISH findings. Patients were excluded if they had localized breast cancer, HER2-negative status, or a diagnosis before December 2021 or after June 2024 or if they were treatment-naïve.

We identified a cohort of 100 patients meeting the inclusion criteria using the convenience sampling method. All patients had an Eastern Cooperative Oncology Group (ECOG) performance status of 0 or 1, with some presenting with cerebral metastases. Patients received T-DXd administered as an intravenous infusion every three weeks at a dose of 5.4 mg/kg of body weight, with dose adjustments (first dose reduction is 4.4 mg/kg, and second dose reduction is 3.2 mg/kg), based on individual tolerance. Treatment was continued until evidence of disease progression or the occurrence of intolerable side effects. Responses to treatment were evaluated using the Response Evaluation Criteria in Solid Tumors (RECIST). Progression-free survival (PFS) and overall survival (OS) were analyzed using Kaplan-Meier survival curves. Toxicity was graded according to the Common Terminology Criteria for Adverse Events (CTCAE). All statistical analyses were conducted using SPSS IBM software version 26 (IBM Corp., Armonk, New York, NY, US).

## Results

Among the cohort of 100 patients who underwent T-DXd therapy in settings beyond the first line for unresectable or metastatic cases, 82 patients (82%) exhibited HER2-positive histology, while 18 patients had low HER2 expression with IHC +1 and +2 (n = 12 and n = 6, respectively). Hormonal receptors (estrogen and/or progesterone) were positive in 62 (62%) patients and negative hormonal receptors in 36 (36%). Sixty-four patients (64%) were in the age group of 40-60 years, with the median age being 47.5 years. All patients had an ECOG performance status of 0-1 at baseline. The predominant histology among these patients was grade 3 invasive ductal carcinoma (IDC). Of all the patients, only one was male, and the rest were female. Fifty-seven patients (57%) had high Ki-67 proliferation of >20%, while only eight patients (8%) had a proliferation rate of ≤20%, and for the rest of the patients, the Ki-67 proliferation rate was unknown.

**Figure 1:**
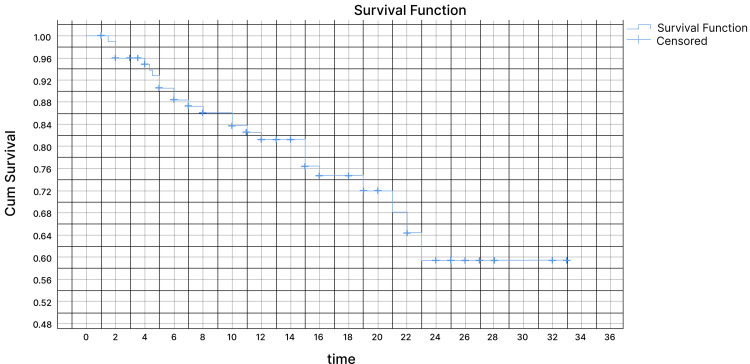
Kaplan-Meier curve for OS OS: overall survival

**Figure 2:**
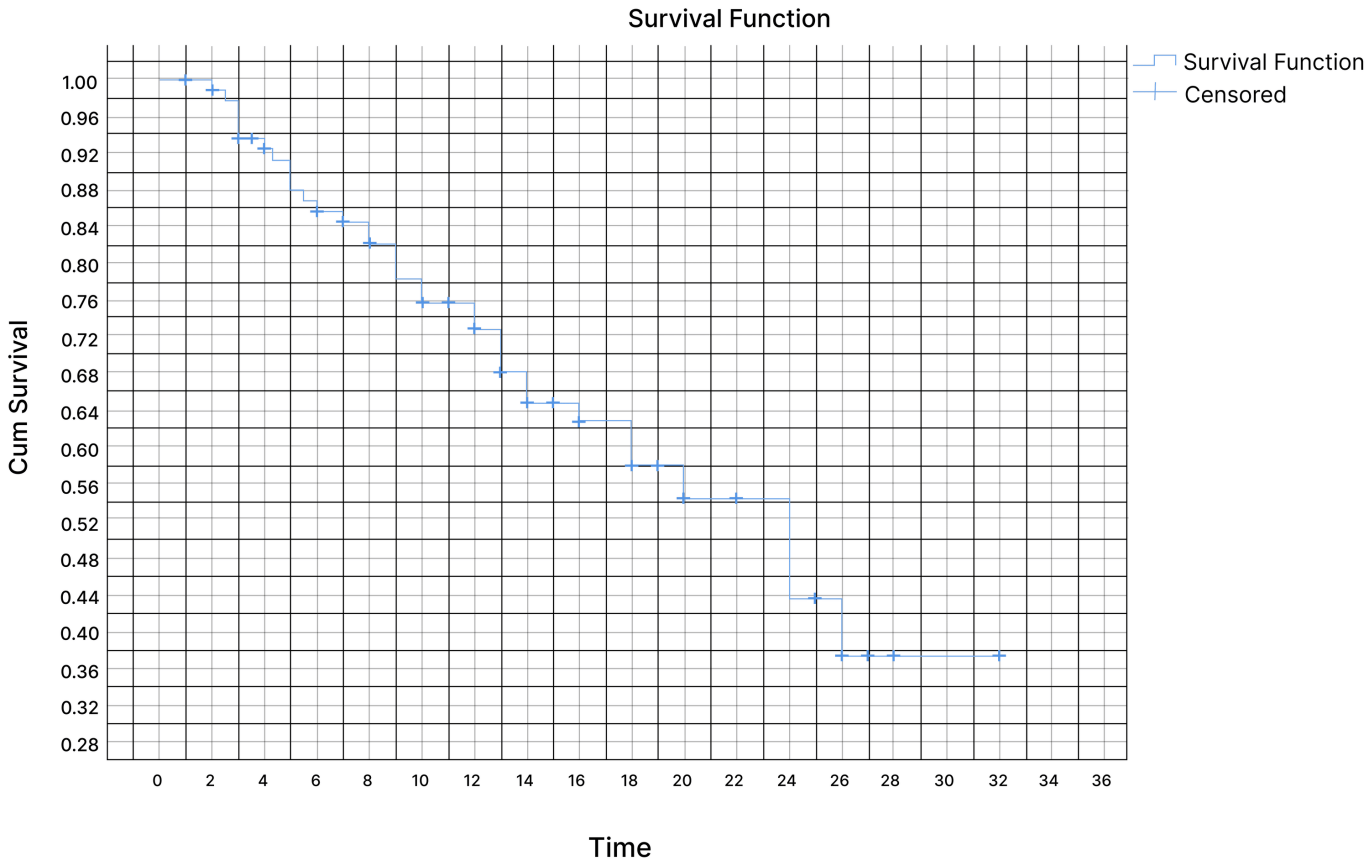
Kaplan-Meier curve for PFS PFS: progression-free survival

Out of the total patients, 84 patients (84%) showed a clinical benefit to the treatment. Among these patients, 10 (10%) had a complete radiological response, 65 patients (65%) had a partial radiological response, and nine patients (9%) had stable disease. However, 10 patients (10%) showed no response to the treatment or clear disease progression. At the time of data collection, 61 patients (61) were alive, 25 patients (25%) had passed away, and 14 patients (14%) were no longer being followed up. During the follow-up period ranging from one to 32 months, with a median of 15 months, it was observed that 66 patients (66%) had metastasis disease to the bone, while 31 patients (31%) had brain involvement. Among the patients, 69% received T-DXd in the second- and third-line setting (34% and 35%, respectively), while the remaining patients received T-DXd in the fourth line (14%), fifth line (13%), and sixth line (5%). In our retrospective study, the Kaplan-Meier curve in Figure 1 shows the OS at one year was 81.5% and 59.5% at 24 months at 95% CI. The study time was not long enough to calculate the median survival rate. Looking at our disease progression data, 35 patients had a progression of the disease. The Kaplan-Meier curve for the estimation of PFS (as shown in Figure 2) showed 73% had PFS at 12 months and 43.5% at 24 months (95% CI). The median PFS was 24 months (95% CI).

The most frequent adverse events of any grade in the T-DXd group were hematological toxicities seen as neutropenia and thrombocytopenia in 15 (15%) and 12 (12%) patients, respectively, classified into grades 1 to 4 as shown in Table 1. The following common side effect was gastrointestinal disturbances, including nausea, vomiting, and abdominal pain in 11 patients (11%) ranging from grade 1 to 2. Deranged liver enzymes were significantly noticed in nine patients (9%). Grade 5 drug-induced hepatic toxicity was reported in one patient (1%). Three patients reported experiencing fatigue of grade 2. Notably, six cases (6%) exhibited grade 3 or higher toxicities, while the remaining toxicities were of grades 1 and 2. One patient (1%) developed grade 3 drug-induced pneumonitis after two cycles of starting T-DXd, which took two months to recover. Other treatment-related adverse events included peripheral neuropathy of grades 1 and 2 in five patients (5%), grade 1 blurred vision in two patients (2%), and grade 1 skin rash and headache in one patient (1%). Prophylactic anti-emetics were provided to all patients in our facility. A left ventricular ejection fraction (LVEF) decrease has been observed in one patient with no intervention needed. Dose reduction intervention was necessary for 13 patients (13%) and discontinuation in two patients (2%).

**Table 1:** Adverse events in patients who received T-DXd ILD: interstitial lung disease; T-DXd: trastuzumab deruxtecan

Adverse events	Grades	Patient number
Thrombocytopenia, n = 12 (12%)	Grade 1	3 (3%)
	Grade 2	7 (7%)
	Grade 4	2 (2%)
Neutropenia, n = 15 (15%)	Grade 1	2 (2%)
	Grade 2	11 (11%)
	Grade 3	2 (2%)
Nausea & vomiting, n = 9 (9%)	Grade 1	5 (5%)
	Grade 2	4 (4%)
Hepatotoxicity, n = 9 (9%)	Grade 1	6 (6%)
	Grade 2	2 (2%)
	Grade 5	1 (1%)
Neuropathy, n = 5 (5%)	Grade 1	4 (4%)
	Grade 2	1 (1%)
Fatigue, n = 3 (3%)	Grade 2	3 (3%)
Abdominal pain, n = 2 (2%)	Grade 1	1 (1%)
	Grade 2	1 (1%)
ILD, n = 1 (1%)	Grade 3	1 (1%)
Blurry vision, n = 2 (2%)	Grade 1	2 (2%)
Headache, n = 1 (1%)	Grade 1	1 (1%)
Skin rash, n = 1 (1%)	Grade 1	1 (1%)

## Discussion

Our center has gained significant experience in the use of T-DXd as a treatment for low/positive HER2 MBC in real-world scenarios. This experience has led to a deeper understanding of the similarities and differences between our findings and those reported in the DESTINY trials. It has also revealed the challenges associated with translating the successes of clinical trials into practical clinical settings. The publication of this study aims to share these findings and collective knowledge with the medical community, and we remain committed to advancing our understanding of this treatment for the benefit of our patients.

The phase II DESTINY-Breast01 trial showed promising results for T-DXd in this patient population and was granted accelerated approval by the FDA [24]. In the subsequent DESTINY 03 phase III trial, patients were randomly assigned to receive either T-DXd or the physician's treatment choice in HER2-positive MBC [25]. The results showed that T-DXd significantly improved median PFS to 17.8 months compared to 6.9 months with the physician's choice, demonstrating strong efficacy in a population with limited treatment options [25]. In the DESTINY 04 trial, patients with HER2-low MBC who had previously received one or two lines of chemotherapy were randomly assigned in a 2:1 pattern to receive either T-DXd or their physician's choice of chemotherapy [26]. The study showed that the OS and median PFS were 23.4 and 9.9 months in the T-DXd group and 16.8 and 5.1 months in the physician's choice group [26]. However, grade 3 or higher adverse events occurred in 52.6% of the patients who received T-DXd and 67.4% of those who received the physician's choice of chemotherapy [26]. Additionally, drug-related interstitial lung disease (ILD) or pneumonitis occurred in 12.1% of the patients who received T-DXd, with 0.8% experiencing grade 5 events [26]. More recently, DESTINY-Breast06 [27] has shown T-DXd as an effective therapeutic agent for both HER2-positive and HER2-low MBC. The trials have demonstrated impressive efficacy and a manageable safety profile across different patient groups [27].

In our center, we have used T-DXd beyond the first line of treatment for unresectable or metastatic cases and found a substantial treatment response. Around 84 patients (84%) showed significant clinical benefit, somewhat higher than the outcomes reported in the DESTINY-Breast01 trial. However, the real-world setting introduces variables only sometimes present in a controlled clinical trial environment, such as patient comorbidities, varied prior treatments, and adherence to therapy, which can affect treatment outcomes.

Drug-induced ILD or pneumonitis is a serious condition marked by inflammation and fibrosis of the lung interstitium. The diagnosis of drug-induced ILD/pneumonitis involves ruling out other conditions, requiring a detailed patient history, physical exam, laboratory tests, and radiographic imaging. Pulmonary toxicity was closely monitored in our center through clinical assessments conducted at every patient visit, which occurred every three weeks. If clinical suspicion arose, further evaluation was performed using high-resolution CT (HRCT). Additionally, lung fields were routinely assessed as part of the scheduled three-month CT chest evaluations, ensuring comprehensive monitoring for ILD or other pulmonary complications. The incidence and management of T-DXd-induced ILD/pneumonitis are not well documented. This lack of data may stem from inconsistent terminology and limited clinical experience in diagnosing and treating this condition. In our cohort, the most common adverse events were hematological, consistent with the trials where myelosuppression was a notable side effect. However, our study reported during the treatment period only one patient with ILD, a severe adverse event well noted in the DESTINY trials.

A meta-analysis and systematic review examined 1,193 patients across 14 studies from various countries and regions with different types of advanced solid malignancies for T-DXd-induced ILD. The overall incidence of all-grade ILD/pneumonitis cases, as adjudicated by an independent committee, was 11.40%, with most cases being grade 1 or grade 2 [28]. Notably, three studies in this review did not report any ILD cases during the treatment period, and one study reported one case only [28].

The exact mechanism of T-DXd-induced ILD is still unknown but is likely associated with the cytotoxic agent, deruxtecan. Understanding drug-associated lung injury mechanisms is limited compared to other tissues like the liver, and there are no specific markers to differentiate drug-associated ILD from other pathological processes. It is a multi-step process, with one key initiating factor likely being the apoptosis of non-neoplastic type I and II pneumocytes. Mitochondrial-mediated apoptotic pathways, which are activated in lung tissues of patients with idiopathic interstitial pneumonia, might play a role in the disease's pathophysiology [29].

The significant differences in ILD risk observed in our study may be influenced by patient comorbidities and prior treatment histories, such as recent exposure to immunotherapy, which can increase the risk of ILD/pneumonitis. Additionally, genetic and ethnic background differences affecting genomics and proteomics may contribute to susceptibility. For example, genetic deficiencies in drug metabolism enzymes, such as N-acetyltransferase, responsible for metabolizing the anti-tuberculosis drug isoniazid, can lead to higher susceptibility to certain drugs. A previously published anecdotal case highlighted a genetic deficiency associated with lung toxicity [28,29].

All patients receiving T-DXd should be monitored for ILD/pneumonitis symptoms. If patients develop a cough, dyspnea, fever, or new respiratory symptoms, an immediate evaluation for ILD/pneumonitis should be initiated promptly, including radiographic imaging and potentially a pulmonologist consultation. Seven studies from the meta-analysis review discussed guidelines for managing suspected T-DXd-induced ILD/pneumonitis adverse events [28]. In these studies, lung toxicity cases were managed through dose interruption, reduction, or discontinuation, corticosteroids, and supportive care. According to these guidelines, T-DXd treatment was interrupted if ILD/pneumonitis was suspected, and corticosteroids were started immediately [28]. If the adverse event was confirmed as grade 1, T-DXd was restarted only after full resolution. The dose was maintained if resolved within 28 days of onset but reduced by one level if resolved after more than 28 days. For grade 2, 3, or 4 events, T-DXd was permanently discontinued. Other studies suggest a rechallenge of treatment in patients with toxicity grade 2 and with a rapid recovery period [28,30].

HER2-low breast cancer, characterized by an IHC score of 1+ or 2+ with negative ISH, represents a distinct subgroup with important clinical and prognostic implications. Unlike HER2-positive tumors, which respond to established HER2-targeted therapies, HER2-low tumors exhibit unique biological behavior, particularly concerning hormone receptor (HR) status. The development of ADCs like T-DXd has introduced a promising therapeutic avenue for this subgroup, warranting further investigation to refine treatment strategies. In our study, we evaluated six patients with HER2-low disease who received T-DXd with the same inclusion criteria aligned with DESTINY-Breast06 [27]. All patients achieved a partial response, with a median PFS of 11 months. Notably, the median PFS in DESTINY-Breast06 was 13.2 months (95% CI) after 18 months of follow-up [27], which is comparable to our results. Additionally, no significant differences in toxicity profiles were observed between HER2-low and HER2-positive subgroups. While the small sample size limits definitive clinical conclusions, these findings suggest a potential benefit of administering T-DXd in earlier treatment settings for HER2-low breast cancer, reinforcing the need for further research in this evolving landscape.

## Conclusions

Our real-world experience confirms the effectiveness of T-DXd in treating HER2-positive and HER2-low MBC, similar to the efficacy seen in the DESTINY trials. Despite the challenges of clinical practice, T-DXd has shown substantial disease control with a manageable safety profile, highlighting its importance in the treatment of MBC. ILD/pneumonitis is a well-documented and serious adverse event associated with T-DXd. In our study, the incidence rate was much lower than reported in the DESTINY trials. Patient comorbidities, as well as genetic and ethnic backgrounds, may contribute to the variations in ILD incidence. Until further data is available, it may be reasonable to continue using the 12-weekly assessment scans to monitor patients for ILD. The key is to clinically monitor patient symptoms and initiate investigations as needed. Further research is necessary to identify the risk factors and underlying pathophysiology of T-DXd-induced ILD/pneumonitis to prevent its occurrence and to determine the optimal imaging frequency for monitoring ILD.
